# HIV testing practices among black primary care physicians in the United States

**DOI:** 10.1186/1471-2458-13-96

**Published:** 2013-02-02

**Authors:** Eric Y Wong, Wilbert C Jordan, David J Malebranche, Lori L DeLaitsch, Rebecca Abravanel, Alisha Bermudez, Bryan P Baugh

**Affiliations:** 1 Janssen Therapeutics, 1125 Trenton-Harbourton Road, Titusville, New Jersey, USA; 2 OASIS Clinic, 1807 East 120th Street, 90059, Los Angeles, California, USA; 3 Emory University Division of General Medicine, 49 Jesse Hill Jr. Drive, Suite 413, 30303, Atlanta, Georgia, USA; 4 Added Value Cheskin, 255 Shoreline Drive, Suite 350, 94065, Redwood Shores, California, USA

**Keywords:** HIV, Testing, Survey, Black/African-American, Physicians

## Abstract

**Background:**

The Centers for Disease Control and Prevention recommends routine HIV testing in all healthcare settings, but it is unclear how consistently physicians adopt the recommendation. Making the most of each interaction between black physicians and their patients is extremely important to address the HIV health disparities that disproportionately afflict the black community. The goal of this survey-based study was to evaluate the perceptions and practices of black, primary care physicians regarding HIV testing.

**Methods:**

A physician survey was administered at the 2010 National Medical Association Annual Convention, via online physician panels, and by email. Physician eligibility criteria: black race; practicing at least 1 year in the US; practice comprised of at least 60% adults and 20% black patients. Contingency tables and ordinary least squares regression were used for comparisons and statistical analyses. A Chi-square test compared percentages of physicians who gave a particular response and a *t*-test compared the means of values provided by physicians.

**Results:**

Physicians over-estimated HIV prevalence and believed that HIV is a crisis in the black community, yet reported that only 34% of patients were HIV tested in the past year. Physicians reported that 67% of those patients tested did so due to a physician recommendation. Physicians who were younger, female, obstetricians/gynecologists, and had a higher proportion of black, low-socioeconomic status, and Medicaid patients reported higher testing rates. Most testing was risk-based rather than routine, and three of the five most commonly reported barriers to testing were related to disease stigma and perceived value judgments. Physicians reported that in-office patient informational materials, increased media attention, additional education and training on HIV testing, government mandates requiring routine testing, and accurate pre-packed tests would most help them test more frequently for HIV.

**Conclusions:**

In this sample of black, primary care physicians, HIV testing practices differed according to physician characteristics and practice demographics, and overall reported testing rates were low. More physician education and training around testing guidelines is needed to enable more routine testing, treatment, and long-term management of patients with HIV.

## Background

In 2006, the Centers for Disease Control and Prevention (CDC) recommended universal, opt-out testing for human immunodeficiency virus (HIV) in all healthcare settings for individuals aged 13–64 years (unless local prevalence of undiagnosed HIV infection is <0.1%) [1]. It is estimated that about 1-in-5 HIV-infected individuals in the United States (US) are unaware of their status, and the CDC guidelines were put in place to increase the number of early diagnoses, improve linkage to care, and encourage individuals who test positive to modify their behaviors to reduce transmission [2-4]. Among persons initially diagnosed with HIV in 2008, one-third received an acquired immune deficiency syndrome (AIDS) diagnosis within 12 months [5]; these represent late diagnoses that are missed opportunities for treatment and prevention. And although disproportionately affected populations such as minorities and men who have sex with men (MSM) were typically tested for HIV more frequently than other groups, targeted HIV testing strategies such as these were insufficiently picking up the fluid sexual networking and condom use behaviors of all sexually active adults [6,7]. The CDC recommends that sexually active patients be screened for HIV at least once, and high-risk individuals be tested for HIV annually, but a 2008 study in 21 major US cities found that about 40% of MSM had not been tested for HIV in the past year [8]. From 2006 to 2009, overall HIV incidence did not change significantly in the general population or among specific racial/ethnic groups, but there was a 21% increase in incidence among people aged 13–29 years, driven primarily by a 34% increase among young MSM and 48% increase among young, black MSM [9]. The black community bears a disproportionate burden of HIV, accounting for only 13% of the population but more than 50% of all HIV diagnoses in 2009 [6,10]. Despite this, only about 3-in-5 black people have ever been tested for HIV [7].

It is unclear how consistently physicians adopt the CDC testing guidelines. Previous studies suggest that HIV testing frequency could be improved if physicians increased the number of recommendations that they make to patients [11-13]. Many black patients may preferentially seek medical treatment from black physicians, and black physicians are more likely to practice medicine in predominantly black communities, but black physicians account for only about 3% of physicians in the US [14,15]. Making the most of each interaction between black physicians and their patients is extremely important to address the HIV health disparities that afflict the black community [16]. To better understand some of the barriers to and facilitators of HIV testing in the black community, the characteristics of physicians who represent the gatekeeper to HIV testing recommendations should be studied and characterized. For the purpose of this study, we designed a survey to evaluate HIV testing perceptions, predictors, and barriers among black, primary care physicians in the US according to physician characteristics and physician-reported patient demographics (see Additional file 1).

## Methods

The survey was developed in partnership with the National Medical Association (NMA), the US’s oldest and largest medical association representing more than 30,000 black physicians and their patients, to examine how physician characteristics and practice composition affect HIV testing attitudes and behaviors in the black community. Survey questions were created with input from black physician advisors who test, treat, and manage patients with HIV. This survey-based research activity was reviewed and ethically approved by an NMA executive committee. The study included physicians only; no patients or human subjects were involved.

The 55-question (including demographic and screening questions) survey required an average of 18 minutes to complete and was administered to physicians via two online survey panels (Panel 1, July-August 2010, non-NMA members only; Panel 2, October-November 2010, NMA members), conference intercept surveys at the 2010 NMA Annual Convention (August 2010), and post-conference emails to individuals on the NMA master file (September-October 2010). Physicians who completed the survey at the NMA Annual Convention were excluded from the online panels and post-conference emails.

### Inclusion criteria

Physician respondents were screened for the following and terminated from the survey otherwise: black race; specialty in internal medicine (IM)/general practice (GP), obstetrics/gynecology (OB/GYN), family practice (FP), or emergency room (ER)/urgent care; practicing medicine for ≥1 year in the US; treating primarily adults (≥60% of patients 18 years or older); practice comprises ≥20% black patients.

### Physician characteristics

Included: gender, age, race, years practicing medicine, specialty, NMA membership status, and percentage of patients treated by physicians themselves. The survey also inquired about physicians’ views concerning how proud they are of their black heritage, whether they practice safe sex, and whether they are religious.

### Practice characteristics

Included: setting, type, and size; for-profit, non-profit, or government-affiliated; geographical location.

### Demographics of patient base

Included: gender composition; percentage ≥18 years; percentage black race; socio-economic status (SES); type of insurance coverage; percentage with HIV/AIDS.

### Physician perceptions of HIV

These were evaluated by asking about: severity of HIV today in the general and black populations; prevalence of HIV at the local and state levels; need to test routinely; type of patients recommended to take a test; percentage of patients recommended for testing; primary reasons for recommending a test; frequency physicians themselves are tested.

### Physician-patient communication

This was evaluated by asking about: comfort level raising issue of HIV testing; scenarios in which the issue of HIV testing may be raised; perceived openness of patients to testing; common emotional reactions to a test recommendation; reasons given by patient for refusing or not discussing HIV testing; barriers to recommending a test; factors that would help physicians test more.

### Statistical analysis

Contingency tables and ordinary least squares regression were used for comparisons and statistical analyses (performed on SPSS v.17). A Chi-square test was used to compare percentages of physicians who gave a particular response and a *t*-test compared the means of values provided by physicians.

## Results and discussion

The practices and perceptions of black, primary care physicians who treat predominantly adult, black patients in the US were evaluated in a survey-based research study to identify key barriers and facilitators of HIV testing. There were 502 surveys included in the analysis, out of >34,000 invitations. Response rates were 0.8% [101/12665] for Panel 1, 5% [46/919] for the post-conference emails sent to those on the NMA master file, and 0.6% [124/20463] for Panel 2 (0.6% [44/7261] among NMA members and 0.6% [80/13202] among non-NMA members). There were 231 intercept surveys completed at the NMA Annual Convention. In all, 321 (64%) of the surveys were completed by NMA members. Characteristics of the respondents and their practices are described in Table 1. Physician-reported demographics of patient base: 56% black race; 67% women; 87% ≥18 years of age; 24% on Medicaid (13% uninsured); 31% with low SES (defined as ‘poor’ on the survey); 6% with HIV/AIDS.

**Table 1:** Physician and practice characteristics of respondents who met inclusion criteria (physician reported)

**Parameter, %**^**a**^	**Internal medicine/general practice [n=185, 37% of respondents]**	**Obstetrics/Gynecology [n=130, 26%]**	**Family practice [n=123, 25%]**	**Emergency room/urgent care [n=64, 13%]**	**All specialties [N=502, 100%]**
Female	42	65	54	61	53
Age, years					
<40	29	25	27	25	27
40–49	34	35	27	45	34
≥50	38	40	46	30	39
Years Practicing					
1–5	13	20	14	16	15
6–10	25	18	22	19	22
11–15	20	15	16	22	18
16–20	16	16	10	19	15
21–30	19	23	31	23	24
≥31	6	8	7	2	6
Region in US					
Northeast	25	9	6	22	16
South	55	63	61	56	59
Midwest	12	18	24	14	17
West	8	10	10	8	9
Practice Setting^b^					
Office	50	57	65	8	50
Hospital	29	31	20	64	32
Academia	21	19	14	36	21
Community	21	12	27	11	19
Practice Type^b^					
Private/For-profit	68	76	64	48	67
Non-profit	30	24	32	39	30
Government	8	7	12	22	10
Proud of black heritage	72	82	76	86	77
Practice safe sex	60	57	52	52	56
Religious	43	54	50	34	46

^a^Percentages may sum to >100% due to rounding; ^b^Respondents were allowed to make multiple selections; US, United States.

More physicians perceived the HIV/AIDS epidemic to be a crisis in the black population than in the general US population (55% vs. 14% respectively), and physicians generally severely over-estimated local county and state HIV prevalence to be 13-14% on average (the highest actual local prevalence is approximately 3% in Washington DC [17] and nationwide it is <1% [18]) (Table 2). If physicians believe that up to 14% of the local population has HIV, then HIV testing should be as common and frequent as testing for hypertension (30% in adults ≥18 years old, 2005–2008) [19] or diabetes (8% in all patients, 2010) [20], which occur during nearly every doctor’s visit. Moreover, 80% of physicians responded that HIV testing should be routine and 20% reported that it should be risked based, yet routine HIV testing (55%) was reported as less of a primary reason for testing than risk-based testing (70-89%) (Table 2). Furthermore, physicians reported testing only about one-third (34%) of their patients for HIV in the past year, and much of the testing was risk-based instead of routine. The gap between the percentage of physicians who say that testing should be routine (80%) and those who actually reported testing patients for HIV in the past year (34%) indicates that a disconnect exists which prevents physicians from taking action on their beliefs. Physicians seem to understand that they should test more routinely, but translating that into practice is difficult. Physicians were more likely to test at-risk patients, presumably because it is easier for physicians to point to documented risk factors as justification for recommending a test. In the context of a patient with no discernible risk factors (other than sexual activity), however, physicians may fear appearing judgmental of the patient’s behavior and avoid recommending an HIV test.

**Table 2:** HIV testing perceptions and practices of black, primary care physicians (physician reported)

**Survey Question and Responses**	**% physicians**
“In your opinion, how serious of a problem is HIV today…?”	
“Crisis” in the general population	14
“Crisis” in the black population	55
“What would you estimate is the prevalence of HIV/AIDS to be in the county and state where you practice?”^a^	
State	14
County	13
“When it comes to HIV testing in general, which of the following statements do you agree with most?”	
HIV testing is only necessary for my patients in “high-risk”^b^ groups	20
HIV testing is part of the routine tests I recommend for all of my sexually active patients	80
“What would be the primary reasons you would recommend HIV testing to a patient?”	
Multiple sex partners	89
Injection drug use	85
Sexual assault	83
Suspected prostitution	77
Sexual activity	77
Homosexuality	77
Previous incarceration	70
Routine test	55
“In the past year, what percentage of the patients in your practice has been tested for HIV?”	
All patients	34
Black patients	37
“Have you, yourself, been tested for HIV…?	
In the past year	25
In the past 5 years	50
More than 5 years ago	16
Never	8

^a^The highest actual local prevalence of HIV in the United States is approximately 3% in Washington, DC [17]; ^b^Such as men who have sex with men, injection drug users, and individuals with multiple sex partners.

**Figure 1:**
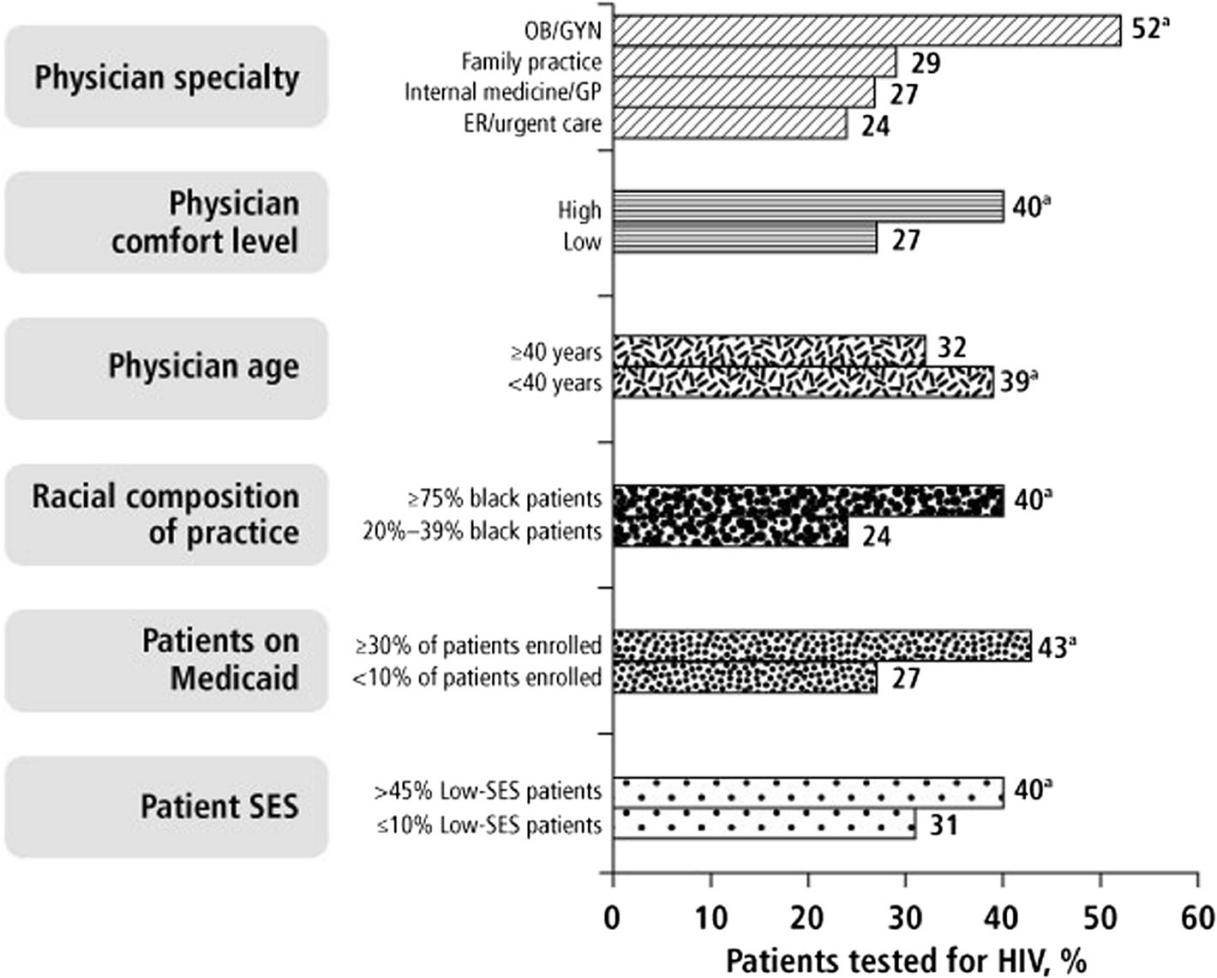
**Percentage of patients reportedly tested for HIV according to key drivers of physician recommendations. **^a^Significantly higher (*P*<0.05) than other group(s) in category; Overall testing rate was 34%; “High” physician comfort level represents those physicians who rated themselves as highly comfortable with raising the issue of HIV with patients who they perceive to be *not* at risk of contracting the virus; Low-SES defined as ‘poor’ on the survey; OB/GYN, Obstetrics/Gynecology; GP, general practice; ER, emergency room; SES, socio-economic status.

Physicians in this sample reported that among patients who were tested for HIV, 67% was due to their or another physician’s recommendation. Physician recommendation for an HIV test was driven most significantly by key factors such as specialty, comfort raising the issue with patients perceived *not* to be at risk for HIV, age of physician, and percentage of black, low-SES, and Medicaid patients in the practice (Figure 1). Significant characteristics (*P*<0.05) that distinguished the “more” and “less” routine testers are listed in Table 3. Ninety-five percent of the “more routine” testers believed that HIV testing should be routine for all patients compared to only 64% of the “less routine” testers, but reported actual testing rates fell far short of these values.

**Table 3:** **Key characteristics of “more routine”**^**a **^**versus “less routine”**^**b **^**testers for HIV in the past year**^**c**^

**More routine testers (n=173) were more likely to:**	**Less routine testers (n=157) were more likely to:**
Be OB/GYN^d^ (47%)	Not be OB/GYN (only 12% are OB/GYN)
Report testing patients as routine practice (74%)	Report testing only patients who have risk factors (39% test routinely)
Be <40 years old (32%; mean age 46 yrs)	Be >40 years old (81%; mean age 49 yrs)
Be women^e^ (60%)	Be men (55%)
Have been tested for HIV themselves in past year (37%)	Not have been tested for HIV themselves in past year (only 16% were tested)
Perceive a higher local prevalence of HIV at the county (16%) and state (16%) levels	Perceive a lower local prevalence of HIV at the county (10%) and state (11%) levels
Have relatively more patients who are:	Have relatively fewer patients who are:
Black (62%)	Black (52%)
Low SES (34%)	Low SES (27%)
On Medicaid (30%)	On Medicaid (18%)
HIV positive (9%)	HIV positive (4%)

^a^Tested more than 25% of patients (>50% on average); ^b^Tested 0-7% of patients (<3% on average); levels of testing were calculated relative to the data distribution of testing rates in which approximately the top one-third of respondents fell into the “more routine” category and the bottom one-third fell into the “less routine” category. ^c^Percent differences between more and less routine testers were significant (*P*<0.05); ^d^OB/GYNs tested more frequently regardless of physician gender; ^e^Female physicians’ testing rates were likely inflated by over-representation of OB/GYNs; OB/GYN, Obstetrics/Gynecology; SES, socio-economic status.

Specialty was the most impactful factor affecting HIV testing rates, with OB/GYNs leading the way with 52% of patients tested within the last year (Figure 1). It may be easier to raise the issue of HIV testing during a routine gynecological exam because testing for HIV can be placed in the context of general screening for sexually transmitted infections since sexual activity is assumed, and women may want to know their HIV status if they are planning to get pregnant or would like to prevent vertical transmission to the child, which align with previous observations of testing practices among OB/GYNs [21,22]. Moreover, guidelines from the CDC, American College of Obstetrics and Gynecologists (ACOG), and many states recommend opt-out HIV testing for pregnant women, and some states even require it [1,23,24]. In contrast to OB/GYNs, physicians in other specialties appear to find it harder to raise the issue of HIV testing in relatively comfortable contexts that would avoid any stigma or perceived judgments associated with HIV; in-office materials such as brochures or posters that educate patients on the importance of getting tested may enable more comfortable physician-patient interactions in these scenarios. While it is true that OB/GYNs see only female patients, the percentage of female patients in physician practices was not a driver of HIV testing due to physician recommendation. Also of note is that although female physicians reported testing a greater percentage of patients for HIV than their male counterparts (38% vs. 29%, *P*<0.05), this may have been due in part to the over-representation of women as OB/GYNs (65%, n=84), and not just a gender-specific predilection. Finally, it should be acknowledged that OB and GYN data were pooled in this analysis, and the percentage of reported patients tested by OBs alone is likely higher than the rate reported for OB/GYNs for the reasons mentioned above related to mandatory testing during pregnancy.

The physician’s comfort level discussing HIV and age was also a predictor of testing frequency (Figure 1) (see Additional file 2). Physicians who rated themselves as highly comfortable with raising the issue of HIV with patients who they perceive to be *not* at risk of contracting the virus tested more frequently than those who did not report being comfortable. Having conversations about HIV routinely rather than only with patients who exhibit risk factors such as multiple sex partners, injection drug use, previous incarceration, or suspected sex work should certainly improve testing rates. Physicians <40 years old tested patients more frequently than those ≥40 years old (39% vs. 32%, *P*<0.05). Because they received medical training during the HIV era (post-1981), younger physicians may have greater education, social comfort, and tolerance with the risk factors associated with HIV [25]. Previous studies have concluded that female and younger physicians obtained sexual histories to a greater extent in primary care settings, and physician discomfort (particularly with opposite gender, very young or old, and homosexual patients) reduced the likelihood of physicians obtaining sexual histories [26-28]. This suggests that physicians, especially older, male physicians, may benefit from more practical clinical strategies and techniques to obtain a sexual history from all patients uniformly.

Physicians with more black (≥75%), low-SES (>45% ‘poor’), and Medicaid (≥30% enrolled) patients in their practice were more likely to test for HIV (Figure 1). These groups are disproportionately affected by HIV and may be perceived to be at higher risk based on traditional risk-based testing recommendations provided by previous public-health and medical organizations. Physicians with more black patients believed that HIV was more of a crisis within the black community than the general US population, and physicians with more black and low-SES patients were more likely to over-estimate HIV prevalence at their county and state levels. Patients on Medicaid receive HIV testing coverage and physicians can use that as a reason to recommend a test.

Physicians with more HIV/AIDS (≥7%) patients tested more than those with fewer HIV/AIDS (≤1%) patients (39% vs. 32%, *P*<0.05), as would be expected.

Among reported barriers to recommending an HIV test, three of the five most common barriers were associated with stigma, patient disclosure, and perceived value judgments; the remaining two reflected competing priorities on physicians’ time (Table 4). Constraints such as shortage of resources or financial considerations (for the physician or patient) were ranked ninth and tenth, respectively. This indicates that physicians’ fear of an uncomfortable interaction with a patient may be a significant impediment to routine testing practices, and proper training (from medical school or continuing education) and sufficient time are important for physicians to routinely initiate the conversations that produce quality sexual histories [26-28].

**Table 4:** **Most commonly reported barriers that limit physicians from recommending HIV testing**^**a**^

**Barriers**	**% physicians**
Patient may perceive the recommendation as accusatory or judgmental	57
Patient wouldn’t want to be identified as HIV positive/worried that people will find out	48
Competing priorities/other needs more urgent	45
Insufficient time with the patient	45
There’s such a stigma associated with HIV, and many doctors don’t want to offend anyone	43

^a^Most frequently mentioned by physicians among their top five barriers according to the survey question: What are the key factors that limit black physicians from recommending HIV testing?

Attitudinal barriers such as physicians’ perceptions of their patients’ reactions to a testing recommendation and reasons for possibly refusing an HIV test differed according to patient characteristics such as race and SES. Physicians reported that black patients were more likely than non-black patients to respond to an HIV testing recommendation with denial (52% vs. 45%), offense (24% vs. 18%), and anger (19% vs. 11%), and give reasons such as “I’m not gay” (55% vs. 47%), “I don’t feel sick” (42% vs. 35%), and “I don’t like needles/giving blood” (37% vs. 31%) for refusing an HIV test (all are *P*<0.05). This may reflect a belief among many physicians that patients may not accurately assess or convey their own risks for acquiring HIV during a clinical encounter. Given that the above responses could be the reasons why many patients refuse HIV testing in clinical settings, this represents an opportunity for developing physician training initiatives on constructive approaches to communicating the importance of testing to patients, particularly given that “physician recommendation” was the most commonly reported driver of HIV testing within this sample. Compared to physicians with fewer low-SES patients, those with more low-SES patients ranked ‘Patient would perceive test recommendation as accusatory or judgmental’ (51% vs. 65%) and ‘Patient wouldn’t want to be identified as HIV-positive’ (41% vs. 59%) as less of a barrier to HIV testing and ‘Physicians lack resources to treat patients with HIV/AIDS’ (27% vs. 17%) and ‘Physicians are ill-equipped to deal with emotional reaction’ (29% vs. 17%) as more of a barrier to testing (all are *P*<0.05)

Only 25% and 50% of physicians reported that they were tested for HIV themselves within the past 1 and 5 years, respectively, and 8% reported having never been tested for HIV themselves (mostly males [14% vs. 4% females, *P*<0.05] and physicians ≥55 years old [17% vs. 6% <55 years old, *P*<0.05]) (Table 2). Physicians who were less likely to get tested for HIV themselves were less likely to recommend testing to their patients (Table 3). There may be a correlation between personal and professional HIV testing practices, and physicians who do not view testing as important in their own lives may project that onto their patients.

**Figure 2:**
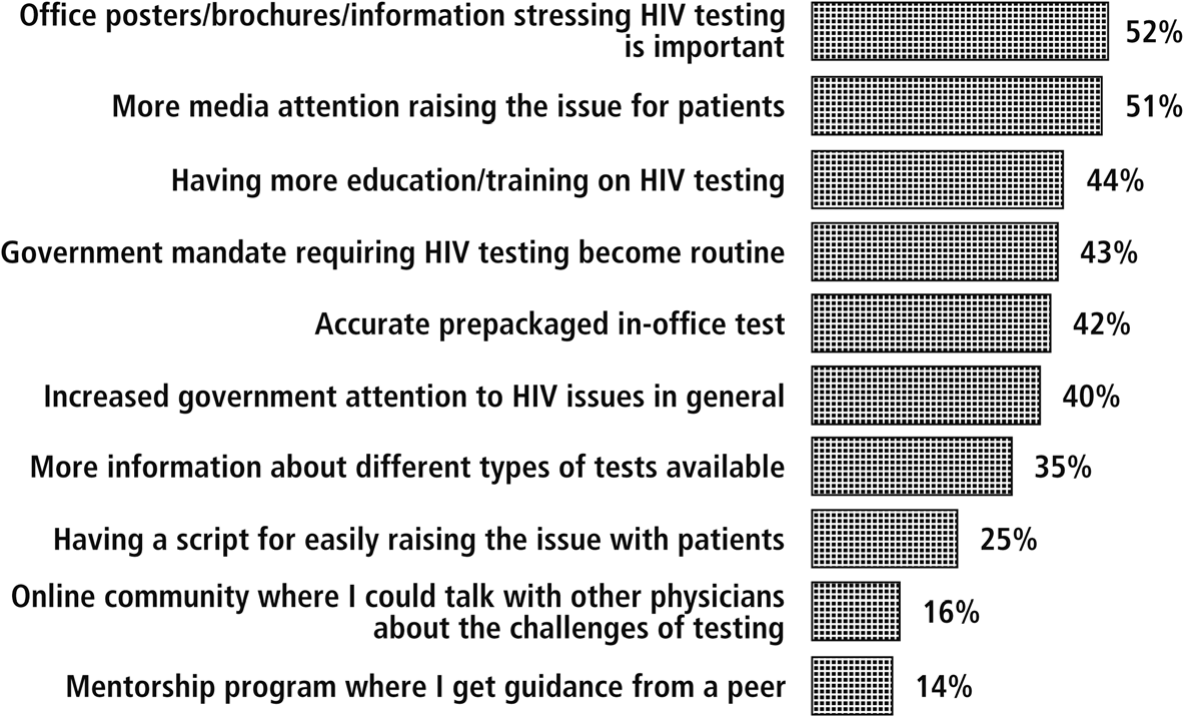
**Factors that would help physicians recommend more HIV testing (percentage who selected factor as important)**^**a**^**. **^a^Based on the survey question: What would help you do more testing? (Factors were selected from a pre-defined list and multiple choices were allowed).

Many of the resources that physicians reported would be important aids to help them test more frequently for HIV, such as in-office patient informational materials, increased media attention, additional education and training on HIV testing, government mandates requiring routine testing, and accurate pre-packed tests, are already available through the CDC, HIV/AIDS societies, and diagnostics manufacturers, but physician awareness and/or access may be limited (Figure 2). The fact that physicians want more in-office informational materials and media attention reflects a desire to encourage patients themselves to raise the question of HIV testing or to increase patients’ comfort levels with the prospect of being tested for HIV. It has been reported that patient-focused rather than physician-focused materials would help primary care physicians more to improve HIV testing practices because physicians felt that resources facilitating communication with patients about HIV are more important than materials that enable physicians to be more proficient in treating HIV patients [29]. Government mandates for routine testing would also lessen the burden on physicians to bring up the issue of testing by providing them with a non-judgmental reason for recommending an HIV test. In fact, many states still have statutes that are inconsistent with CDC guidelines regarding routine, opt-out testing for HIV in all healthcare settings [30]. However, physicians should not place the responsibility of communicating about HIV testing on the patients alone. Those black, MSM, and low-SES patients who need testing the most are often already burdened with social oppressions and these patients may look to physicians to be a source of relief and understanding [31].

This study has several limitations. Firstly, there is the potential for bias associated with volunteering to take the survey, recall, self-reporting, and overestimating or skewing certain responses to exhibit favorable, positive practices. Secondly, the small number of completed surveys (502) relative to the number of invitations distributed (>34,000), the high percentage of respondents who were NMA members, and incomplete geographical representation may mean that the data are not representative of all black, primary care physicians in the US. Thirdly, responses were physician-reported with no patient responses with which to compare, and no overall response rate was reportable due to an unknown response rate for the intercept surveys. Fourthly, some subgroup data may be confounded since OB and GYN data were combined, and OBs differ from all other specialties given their HIV testing requirements. Finally, there is little comparative data on the HIV testing attitudes and behaviors of physicians of other races and ethnicities to make meaningful comparisons using this study’s results. Despite these limitations, this is one of the first studies to examine the reported practices of black physicians with regard to HIV testing.

## Conclusions

Given that black physicians are under-represented in the US medical community but are more likely to practice in the predominantly black communities that are disproportionately affected by HIV, they play an extremely important role in the targeted healthcare goals of implementing routine HIV testing, early access to treatment for those with HIV, and long-term management of HIV care. This study suggests that more education and training is needed to improve the communication of sexual histories during clinical encounters so that both sides are more comfortable discussing topics such as HIV testing and sexual practices in order to diagnose patients with HIV sooner and place them into care. Training efforts and health policies should focus on increasing physician awareness of CDC guidelines calling for routine HIV testing of all sexually active adults, teaching physicians innovative and effective approaches to obtaining a sexual history, and empowering patients to proactively seek HIV testing on their own. Moreover, meaningful collaborations among government agencies, private institutions, community HIV/AIDS organizations and leaders of the black community will be needed to launch coordinated efforts to achieve these goals. As HIV testing practices improve, more patients will enter into care for HIV, and these groups will need to work together to address any deficiencies in resources for treating and supporting these patients. Future studies should evaluate HIV testing practices among physicians of other races and specialties, other healthcare providers such as nurses, nurse practitioners and physician assistants, and how new diagnostic options such as at-home HIV tests will impact community HIV testing practices, and ultimately, patient health outcomes.

## Abbreviations

CDC: Centers for Disease Control and Prevention;HIV: Human immunodeficiency virus;US: United States;AIDS: Acquired immune deficiency syndrome;MSM: Men who have sex with men;NMA: National Medical Association;GP: General practice;OB: Obstetrics;GYN: Gynecology;FP: Family practice;IM: Internal medicine;ER: Emergency room;SES: Socio-economic status;ACOG: American College of Obstetrics and Gynecology

## Competing interests

EYW, LLD, and BPB are employees of Janssen Therapeutics. WCJ and DJM have served as consultants and speakers for Janssen Therapeutics. RA and AB declare no competing interests. This study was funded by Janssen Therapeutics.

## Authors’ contributions

EYW made substantial contributions to the interpretation of data and drafted the manuscript. WJC, DJM, and BPB made substantial contributions to the conception of study design and revised the manuscript critically. LLD made substantial contributions to the interpretation of data and revised the manuscript critically. RA and AB made substantial contributions to the acquisition and statistical analysis of data, and revised the manuscript critically. All authors read and approved the final manuscript.

## Pre-publication history

The pre-publication history for this paper can be accessed here:

http://www.biomedcentral.com/1471-2458/13/96/prepub

## Supplementary Material

Additional file 1 **HIV Testing Survey Questionnaire.** A list of the questions used in the study. Click here for file

Additional file 2 **HIV Testing Survey Regression Table.** Regression analysis showing the key characteristics that affected HIV testing rates. Click here for file
