# Supplementary material for: HIV testing practices among black primary care physicians in the United States
Source: BMC Public Health. 2013 Feb 2;13:96. doi: 10.1186/1471-2458-13-96 (PMC3599058; doi:10.1186/1471-2458-13-96)
Supplement: Additional file 1 — HIV Testing Survey Questionnaire. A list of the questions used in the study. [file 1471-2458-13-96-S1.pdf]

ZIP	What is the zip code of your primary practice?<br><br>Enter 5 digit zip code _________

HON	Physicians practicing in the District of Columbia, Maine and Vermont are not allowed to receive honoraria for participating in studies such as this one. Are you willing to continue with the survey without receiving any honorarium?<br><br>Yes ❑<br>No ❑
S1	Please record your gender.<br><br>Male ❑<br>Female ❑
S2	In what year were you born?<br><br>Enter age in years _________
S3a	Which of the following best describes your ethnic background?<br><br>Black/African American ❑<br>Asian ❑<br>Caucasian ❑<br>Other ❑
S3b	Are you of Hispanic or Latino origin or ancestry?<br><br>Yes ❑<br>No ❑
S4	First, we would like to know about your practice. For how many years have you been practicing medicine? If you have been practicing for less than one year, please enter “0.”<br><br>Enter number of years _________

S5 What is your specialty or type of practice?

Emergency Room/Urgent Care	❑
GP/General Practitioner	❑
Family Practitioner	❑
Internal Medicine	❑
OB/GYN	❑
Pediatrics	❑
Other Specialty	❑

S6 Which of the following describe where you practice? Please select all that apply.

Academic-based practice	❑
Office-based practice	❑
Hospital-affiliated practice	❑
Hospital-based practice	❑
Community-based clinic	❑
Specialized clinic	❑

S7 How would you characterize your practice (s)? Please select all that apply.

Private/for-profit	❑
Non-profit	❑
Government-affiliated (VA, military, state/city/county/district hospital, etc.)	❑

S8a Are you employed by the US Federal Government or any State or Local Government?

Yes ❑
No ❑

S8b Are you covered by the standards applicable to federal, state, or local government employees and officials, including part-time employees and government hospital staff?

Yes ❑
No ❑

S8c Are you a member of a P&T Committee of a health plan, pharmacy benefit manager, or other health care provider or payer covered by the standards applicable to federal, state, or local government employees and officials, including part-time employees and government hospital staff?

Yes ❑
No ❑

S8d Does your participation in this study comply with applicable ethics rules of the P&T Committee, including standards for conflict of interest, disclosure and recusal?

Yes ❑
No ❑

S9 Are you a member of the National Medical Association (NMA)?

Yes ❑
No ❑

S10 What percentage of your patients are aged 18 or over?

Enter percentage _________

S11 What percentage of your patients are Black/African American (regardless of country of origin)?

Enter percentage _________

MAIN QUESTIONNAIRE

Physician's Practice & Patient Base

Q1a/b How large is your practice? Please enter (a) the total number of patients in your practice, and then (b) the number of patients you personally care for. If you do not know the exact number, please give your best estimate.

Enter # of patients in practice ___

Enter # of patients you personally care for ___

Q2 Do you see more men or women in your overall practice? Out of your total patient population, what percentage of Black /African American patients are men and women? Please enter the percentage of men and women that you see.

	All patients	Black/African American patients
% men	________	________
% women	________	________
Total (Must add to 100)	________	________

Q3 a. How would you describe the socio-economic status of your patient base, in total?
b. How would you describe the socio-economic status of your Black/African American patients?

	Total Patient Base	Black/African American Patient Base
% Poor	________	________
% Working Class	________	________
% Middle Class	________	________
% Upper Middle Class/ Wealthy	________	________
Total (Must add to 100)	________	________

Q4 a. How would you describe your patient base (in total) in terms of insurance/payer type?
b. How would you describe your Black/African American patient base in terms of insurance/payer type?

	Total Patient Base	Black/African American Patient Base
% Medicare	______	______
% Medicaid	______	______
% Department of Defense	______	______
% VA	______	______
% Private insurance	______	______
% Payers such as Kaiser/other staff models	______	______
% Other insurance	______	______
% Uninsured	______	______

Q5 What percentage of your patients are HIV positive or have AIDS?

% HIV/AIDS _________

HIV-Specific – Testing & Awareness of CDC Guidelines

We are now going to focus on HIV.

Q6 In your opinion, how serious of a problem is HIV today…

	Crisis	Very serious	Somewhat serious	Not particularly serious	Not at all serious
In general in the US	❑	❑	❑	❑	❑
In the Black/African American community in the US	❑	❑	❑	❑	❑

Q7 What would you estimate is the prevalence of HIV/AIDS to be in the county and state where you practice…

% people with HIV/AIDS in the **county** where you practice ____

% people with HIV/AIDS in the **state** where you practice _____

Q8 When it comes to HIV testing in general, which of the following statements do you agree with most? Please select only one response.

HIV testing is only necessary for my patients in "high risk" groups (such as men who have sex with men, injection drug users, and individuals with multiple sex partners)	❑
HIV testing is part of the routine tests I recommend for all of my sexually active patients	❑

Q9 For which of the following patients would you typically recommend HIV testing? Please select all that apply.

Patients in "high-risk" groups, such as men who have sex with men, injection drug users, and individuals with multiple sex partners	❑
Patients aged 13-64 in areas where HIV prevalence of undocumented HIV infection is greater than 0.1%	❑
Patients seeking treatment for STDs	❑
Patients initiating treatment for Tuberculosis	❑
Pregnant women or women trying to become pregnant	❑
Patients who have been incarcerated in the past 5 years	❑
All patients who are sexually active	❑

Q10 Which of the following are required in your state when testing a patient for HIV? Please select all that apply.

Separate written consent for the HIV test	❑
Pre-test counseling	❑
Post-test counseling	❑
Don't know	❑

Q11 In the past year, what percentage of your patients in your practice (total patient base) have been tested for HIV? What about your Black/African American patients?

% total patient base _____

% Black/Af-Am patient base tested _____

Q12 Of those that have been tested, what percentage of your patients took the test because of a physician recommendation (whether you or someone else), and what percentage requested the test themselves? Please answer for your patients in total, and then for your Black/African American patients.

	All patients	Black/African American patients
% tested due to physician recommendation	_______	____________________
% patient request for test	_______	____________________

Q13 Of those you have recommended for HIV testing, what percentage of your patients (total patient base) has actually been tested? What about your Black/African American patients?

% total patient base who have been tested after recommendation	_____
% Black/Af-Am patient base who have been tested after recommendation	_____
Have never recommended HIV testing for any of my patients	_____

Q14 What would be the primary reasons you would recommend HIV testing to a patient? Please select all that apply.

Routine test	❑
Sexual activity	❑
Multiple sex partners	❑
Injection drug use	❑
Homosexuality	❑
Previous incarceration	❑
Suspected prostitution	❑
Sexual assault	❑
Other (Specify: ______)	❑

Q15 What are the key factors that limit Black/African American physicians from recommending HIV testing? Please choose the top five factors, and assign them a ranking, where 1 is the most important factor.

	Ranking Of Top 5 Factors *(1 is most important)*
Lack of need; very few patients are at risk of HIV—there's no need to test	___
It's difficult to address the issue/start the conversation with patients	___
There's such a stigma associated with HIV, and many doctors don't want to offend anyone	___
Ill-equipped to deal with the emotional reaction of a positive result	___
Patient may perceive the recommendation as accusatory or judgmental	___
Patient doesn't want to spend more time in the doctor's office	___
Patient wouldn't want to be identified as HIV-positive/worried that people they know will find out	___
State requirements for pre or post-test counseling/Lack of time/resources for pre or post-test counseling	___
Insufficient time with the patient	___
Language barrier	___
Competing priorities/other needs more urgent	___
Inadequate reimbursement	___
Patient cannot afford it	___
Concern about patient follow-up	___
Don't want the patient to stop seeking medical care	___
Lacking the resources to treat patients with HIV/AIDS	___
Lacking the proper education/training to address HIV/AIDS	___

Q16 Have you, yourself, been tested for HIV…?

- In the past year ❑
- In the past 5 years ❑
- Ever ❑
- Never been tested ❑

Q17 How comfortable would you say you are with raising the issue of HIV testing with each of the following patient types?

Not at all comfortable						Extremely comfortable
1	2	3	4	5	6	7

a. Black/African American men ____

b. Black/African American women ____

c. Non Black/African American men ____

d. Non Black/African American women ____

e. Patients you perceive to be at risk for HIV ____

f. Patients you DO NOT perceive to be at risk for HIV ____

Q18 We are interested in how you might raise the subject of HIV testing with different types of patients. How would you raise the issue with Black/African American men? How about with Black/African American women? Please answer for both patient types listed below. If you do not typically see the type of patient in your practice, please check “Do not see this type of patient.”

	Black/African American men	Black/African American women
Bring it up during patient intake	❑	❑
Bring it up during annual physical / or gynecological exam	❑	❑
Bring it up when treating an STD	❑	❑
Just say it’s part of a routine exam	❑	❑
When doing other blood work	❑	❑
Raise during urgent care	❑	❑
Whenever the patient has an open wound/bleeding	❑	❑
In cases of sexual assault	❑	❑
When patient seeks help for injection drug use	❑	❑
Do not see this type of patient	❑	❑

Q19 Now please think about how you might raise the subject of HIV testing with Non Black/African American patients. How would you raise the issue with Non-Black/African American men? How about with Non-Black/African American women? Please answer for both patient types listed below. If you do not typically see the type of patient in your practice, please check "Do not see this type of patient."

	Non Black/ Af. Am. men	Non Black/ Af. Am. women
Bring it up during patient intake	❑	❑
Bring it up during annual physical / or gynecological exam	❑	❑
Bring it up when treating an STD	❑	❑
Just say it's part of a routine exam	❑	❑
When doing other blood work	❑	❑
Raise during urgent care	❑	❑
Whenever the patient has an open wound/bleeding	❑	❑
In cases of sexual assault	❑	❑
When patient seeks help for injection drug use	❑	❑
Do not see this type of patient	❑	❑

Q20 In general, how open would you say the following groups of patients are to HIV testing?

Not at all open						Completely open
1	2	3	4	5	6	7

a. Black/African American men ____

b. Black/African American women ____

c. Non Black/African American men ____

d. Non Black/African American women ____

Q21 What are common emotional reactions to the recommendation for HIV testing among your Black/African American patients? What about among your Non-Black/African American patients? Select all that apply.

	Black/African American Patients	Non Black/African American Patients
Deny need for testing	❑	❑
Anger	❑	❑
Offense	❑	❑
Shock/Disbelief	❑	❑
Calm (trusts doctor knows best)	❑	❑
Questioning reasons for request	❑	❑
Understand need to test/eager to take care of their health	❑	❑
Do not routinely recommend HIV testing	❑	❑

Q22	What are common reasons your **overall patient population** gives for not discussing HIV testing or refusing a test? Please select all that apply.	

I’m not gay	❑
I don’t use drugs/I’m clean	❑
I’m in a monogamous relationship	❑
I’m not at risk for HIV	❑
I don’t like needles/giving blood	❑
I don’t want anyone to find out	❑
What if I test positive?	❑
I don’t feel sick	❑
I always practice safe sex	❑
I’m not having sex now	❑
I’ve only had sex with a few people	❑
Other: Specify ___________	❑

Q23	What are common reasons your <u>Black/African American patients</u> give for not discussing HIV testing? Please select all that apply.	

I’m not gay	❑
I don’t use drugs/I’m clean	❑
I’m in a monogamous relationship	❑
I’m not at risk for HIV	❑
I don’t like needles/giving blood	❑
I don’t want anyone to find out	❑
What if I test positive?	❑
I don’t feel sick	❑
I always practice safe sex	❑
I’m not have sex now	❑
I’ve only had sex with a few people	❑
Other: Specify ___________	❑

Q24	Do you think Black/African American patients are more or less comfortable discussing HIV testing with Black/African American physicians than other physicians?	

Black/African American patients <u>are less comfortable</u> talking to an African American physician	❑
Race doesn’t have anything to do with the comfort level	❑
Black/African American patient are <u>more comfortable</u> talking to an African American physician	❑

Q25	What would help you do more testing? Please select all that apply.	
	Having more education/training on HIV testing	❑
	Having a script for easily raising the issue with patients	❑
	Mentorship program where I could get guidance from a peer	❑
	More information about the different types of tests that are available	❑
	Accurate pre-packaged in-office test	❑
	Office posters/brochures/information stressing that HIV testing is important	❑
	Government mandate requiring that HIV testing become routine	❑
	Increased government attention to the issues of HIV in general	❑
	More media attention raising the issue for patients	❑
	Online community where I could talk with other physicians about the challenges of testing	❑
	Other: Specify ________________	❑
	Nothing	❑

Q26	Compared to three years ago, would you say you…	
	Test more of your patients for HIV	❑
	Test about the same number of patients for HIV	❑
	Test fewer of your patients for HIV	❑

Q27	Over the next three years, do you expect to…	
	Test more of your patients for HIV	❑
	Test about the same number of patients for HIV	❑
	Test fewer of your patients for HIV	❑

Q28a/b Please think about your <u>Black/African American</u> <u>physician peers</u>' perceptions of people who are HIV+… What percentage of your peers would you say are disapproving of HIV + patients because of assumptions about their lifestyle?

	Black/African American Physicians
% disapproving of HIV+ people because of assumptions about their lifestyle	____________________

Q29 Do you think your Black/African American physician peers' perception of people who are HIV+ impacts their openness to testing patients for the virus?

- Yes ❑
- No ❑

HIV-SPECIFIC TREATMENT

Q30 If or when a patient tests positive for HIV, do you typically have a conversation with the patient about HIV?

- Yes ❑
- No ❑

Q31 If or when a patient tests positive for HIV, do you typically ask patients how they think they contracted the disease?

- Yes ❑
- No ❑

Q32 Other than potentially talking to the patient, what are the next steps you take if/when a patient tests positive for HIV? Please select all that apply.

- Refer to an HIV or infectious disease specialist ❑
- Initiate treatment immediately ❑
- Monitor CD4 count and viral load until treatment is necessary ❑
- Schedule counseling/refer to support services ❑
- Discuss ways to keep partner safe ❑
- Encourage patient to ask their partner(s)/children to be tested ❑
- Discuss mother-to-child prevention ❑
- Other: Specify_______________ ❑

Q33 Among your patients that are HIV+, what percentage do you treat yourself for the virus versus refer to a specialist for HIV treatment?

% treat yourself _____

% refer to a specialist/another provider _____

Total (Must add to 100) _____

Q34 What are the reasons for referring HIV+ patients to another provider?

- I lack sufficient knowledge around current drug regimens for HIV ❑
- I am not personally comfortable treating HIV ❑
- Low reimbursement rate for HIV treatment ❑
- I am unable to keep up with all of the research, new treatments and guidelines for treatment HIV ❑
- I don't have the time to deal with such a complicated disease on an ongoing basis ❑
- I don't have the resources to treat HIV (other staff, counseling, support services) ❑
- I believe HIV should be treated by a specialist ❑
- Other: Specify______________ ❑

Q35 What would help you to treat more HIV+ patients yourself?

- Better reimbursement rate for HIV treatment ❑
- Better understanding of current drug regimens for HIV ❑
- Additional training/education around HIV in general ❑
- HIV-specific training included in medical school education ❑
- Mentorship program with an experienced HIV treater ❑
- Online portal or community where I could get information and/or connect with other physicians ❑
- Other: Specify ❑
- Nothing, I have no interest in increasing the treatment of HIV in my practice ❑
- Nothing, I believe HIV should be treated by a specialist ❑

Q36 What are your impressions of current HIV treatment? Please select all that apply.

- HIV is a disease with a clear treatment protocol ❑
- HIV is difficult to treat ❑
- HIV requires a complex treatment regimen ❑
- HIV is a chronic condition if managed properly ❑
- HIV is a time-intensive disease for treating physicians ❑
- HIV requires a lot additional office resources ❑
- HIV is a terminal condition ❑
- None of these ❑

DEMOGRAPHICS

D1 Please tell us about yourself by indicating how well each of following statements describes you and things you may believe.

Does not describe me at all	←==→									Describes me perfectly
0	1	2	3	4	5	6	7	8	9	10

a I am proud of my Black/African American heritage ____

b I am a religious person ____

c I am socially conservative ____

d I always practice safe sex ____

D2 Which of the following best describes your sexual orientation?

- Heterosexual/Straight ❑
- Homosexual/Gay/Lesbian ❑
- Bisexual ❑
- Prefer not to answer ❑
