# Supplementary material for: HIV testing practices among black primary care physicians in the United States
Source: BMC Public Health. 2013 Feb 2;13:96. doi: 10.1186/1471-2458-13-96 (PMC3599058; doi:10.1186/1471-2458-13-96)
Supplement: Additional file 2 — HIV Testing Survey Regression Table. Regression analysis showing the key characteristics that affected HIV testing rates. [file 1471-2458-13-96-S2.pdf]

Regression Results: Drivers of Percentage of Patients Tested Due To Physician Recommendation

Variables In Equation	Unstandardized Coefficients, B	Std. Error	Standardized Coefficients, Beta	P Value
(Constant)	0.912	2.972	---	.759
OB/GYN Specialty	17.435	2.363	0.305	.000
Physician aged less than 40	5.028	2.251	0.089	.026
Percent of black patients	0.140	0.046	0.126	.002
Percent of patients on Medicaid	0.263	0.050	0.216	.000
High Comfort Raising Issue with Those NOT at Risk	5.599	2.044	0.112	.006

R^2=0.23, N of cases=502

Notes: The Dependent Variable is Percentage of Patients Tested Due to Physician Recommendation.
Independent Variable Definitions Are:
Age is a bivariate variable coded0 if over 40, and coded 1 if < 40
Percent of black patients is a scale variable.
Percent of Medicaid patients is a scale variable.
High Comfort Raising Issue with Those NOT at Risk is a bivariate variable, coded 0 if the respondent gives a score of 1-4 and coded as 1 if the respondent gives a score of 5-7 on a self-rating of comfort, where 0 is "not at all comfortable" and 7 is "extremely comfortable."
